# A simple and high-sensitivity method for analysis of ubiquitination and polyubiquitination based on wheat cell-free protein synthesis

**DOI:** 10.1186/1471-2229-9-39

**Published:** 2009-04-06

**Authors:** Hirotaka Takahashi, Akira Nozawa, Motoaki Seki, Kazuo Shinozaki, Yaeta Endo, Tatsuya Sawasaki

**Affiliations:** 1 Cell-Free Science and Technology Research Center, Ehime University, Matsuyama 790-8577, Japan; 2 The Venture Business laboratory, Ehime University, Matsuyama 790-8577, Japan; 3 Plant Functional Genomics Research Group, 1-7-22 Suehiro-cho, Tsurumi-ku, Yokohama, Kanagawa 230-0045, Japan; 4 Gene Discovery Research Group, RIKEN Plant Science Center, 1-7-22 Suehiro-cho, Tsurumi-ku, Yokohama, Kanagawa 230-0045, Japan; 5 RIKEN Genomic Sciences Center, 1-7-22 Suehiro-cho, Tsurumi-ku, Yokohama, Kanagawa 230-0045, Japan

## Abstract

**Background:**

Ubiquitination is mediated by the sequential action of at least three enzymes: the E1 (ubiquitin-activating enzyme), E2 (ubiquitin-conjugating enzyme) and E3 (ubiquitin ligase) proteins. Polyubiquitination of target proteins is also implicated in several critical cellular processes. Although Arabidopsis genome research has estimated more than 1,300 proteins involved in ubiquitination, little is known about the biochemical functions of these proteins. Here we demonstrate a novel, simple and high-sensitive method for *in vitro *analysis of ubiquitination and polyubiquitination based on wheat cell-free protein synthesis and luminescent detection.

**Results:**

Using wheat cell-free synthesis, 11 E3 proteins from Arabidopsis full-length cDNA templates were produced. These proteins were analyzed either in the translation mixture or purified recombinant protein from the translation mixture. In our luminescent method using FLAG- or His-tagged and biotinylated ubiquitins, the polyubiquitin chain on AtUBC22, UPL5 and UPL7 (HECT) and CIP8 (RING) was detected. Also, binding of ubiquitin to these proteins was detected using biotinylated ubiquitin and FLAG-tagged recombinant protein. Furthermore, screening of the RING 6 subgroup demonstrated that At1g55530 was capable of polyubiquitin chain formation like CIP8. Interestingly, these ubiquitinations were carried out without the addition of exogenous E1 and/or E2 proteins, indicating that these enzymes were endogenous to the wheat cell-free system. The amount of polyubiquitinated proteins in the crude translation reaction mixture was unaffected by treatment with MG132, suggesting that our system does not contain 26S proteasome-dependent protein degradation activity.

**Conclusion:**

In this study, we developed a simple wheat cell-free based luminescence method that could be a powerful tool for comprehensive ubiquitination analysis.

## Background

Protein ubiquitination plays a crucial role in numerous cellular processes such as cell growth, regulation of diverse signal transduction and disease [1-3]. The covalent attachment of ubiquitin to protein substrates requires a step-wise cascade of enzymatic reactions. First, ubiquitin is activated by E1 (ubiquitin-activating enzyme, UBA) in an ATP-dependent manner by forming a high-energy thioester-bond between the carboxyl-terminal glycine residue of ubiquitin and a cysteine residue of E1. The activated ubiquitin is then transferred to the core-cysteine residue of E2 (ubiquitin-conjugating enzyme, UBC). Together with an E3 ligase enzyme, ubiquitin is attached via its carboxyl-terminus to an e-amino group of a lysine residue in the target protein. Since E3 binds to both E2 and the target protein, and acts as scaffold between E2 and the substrate protein, the E3 ligase is the major determinant for selecting target proteins for ubiquitination. There is large number of genes encoding E3 ligases in all eukaryotes, and the diversity of E3s is thought to contribute to the substrate specificity of numerous target proteins. E3 ligases are structurally divided into three groups: HECT, RING and U-box [4]. The HECT-type E3 ligase is distinct from the other two ligases in that it forms a thioester-bond with ubiquitin prior to the transfer of ubiquitin to target proteins. The RING-type E3 ligase contains a unique domain similar to the zinc finger motif that mediates protein-protein interactions [5] and is further divided into two classes: one that can function alone and another that forms a complex with other E3 components [4].

Recent studies have shown that attachment of polyubiquitin chains on target proteins linked via lysine-48 of ubiquitin typically leads to degradation by the 26S proteasome [6], whereas linkage via lysine-63 mediates different pathways such as internalization of membrane proteins, activation of signal transduction and DNA damage repair [7]. The formation of lysyl-63-linked polyubiquitin chains is generated by specific combinations of E2s and E2 variants, which are similar to E2s except that they lack core cysteine residues required for E2 activity [8,9]. In addition, ubiquitination of substrates without polymerization, mono-ubiquitination, acts as a sorting signal for protein endocytosis and as a regulation factor for diverse proteins, including histones and transcription factors [10].

In plant, genomic research of the model plant *Arabidopsis thaliana *showed that there are two E1s, 37 E2s and more than 1,300 predicted E3s [11]. Although little is known about protein ubiquitination in plants compared with yeast and mammals, recent studies revealed that the plant ubiquitination pathway is involved in the regulation of morphogenesis, the circadian clock and responding to hormone or pathogen signal molecules [12-15]. Despite the importance of ubiquitination in plants, much of the plant ubiquitination cascade is still unknown because of its complexity and the issues inherent to the use of Arabidopsis plants for biochemical analysis. Although several interactions between E2s and RING type E3s have been demonstrated *in vitro *using recombinant proteins expressed in *Escherichia coli*, these efforts are hampered by the inability to obtain functional protein using conventional methods [16].

With this in mind, we sought to develop a novel *in vitro *method to analyze the ubiquitin pathway genome-wide. The two major obstacles hindering the development of an *in vitro *assay for genome-wide screening are the difficulty of efficiently producing recombinant protein and the inability to detect ubiquitination in a high-throughput fashion. To address the first problem we used the wheat cell-free protein synthesis system, which has been previously reported to produce a wide range of functional Arabidopsis and human proteins [17-19]. Moreover, a collection of RIKEN Arabidopsis Full Length (RAFL) cDNA clones covering about 70% of Arabidopsis genes is available [20]. Using these RAFL clones as templates, recombinant proteins involved in the ubiquitination pathway were expressed in the wheat cell-free system and used for several functional analyses. For screening, conventional detection methods such as immunoblot analysis or radioisotope-labeled proteins are not suitable for the detection of a large number of ubiquitination reactions. Recently, a high-throughput luminescence method to detect protein ubiquitination was reported [21], however this method requires purified protein and creation of specialized vectors to produce proteins. In this study, a novel *in vitro *assay to detect polyubiquitin chain formation was developed using wheat cell-free synthesis and a modified luminescence-based detection method. We demonstrate (1) creation of a simple *in vitro *method to detect polyubiquitination using crude recombinant E3s, (2) discovery of the activity of At1g55530 by screening a RING subgroup in the reported assay, and (3) the polyubiquitination assay in the presence of MG132 demonstrated the absence of 26S proteasome-dependent protein degradation activity in wheat cell-free system.

## Results

### Detection of Polyubiquitin Chains on AtUBC22 E2 enzyme

**Figure 1:**
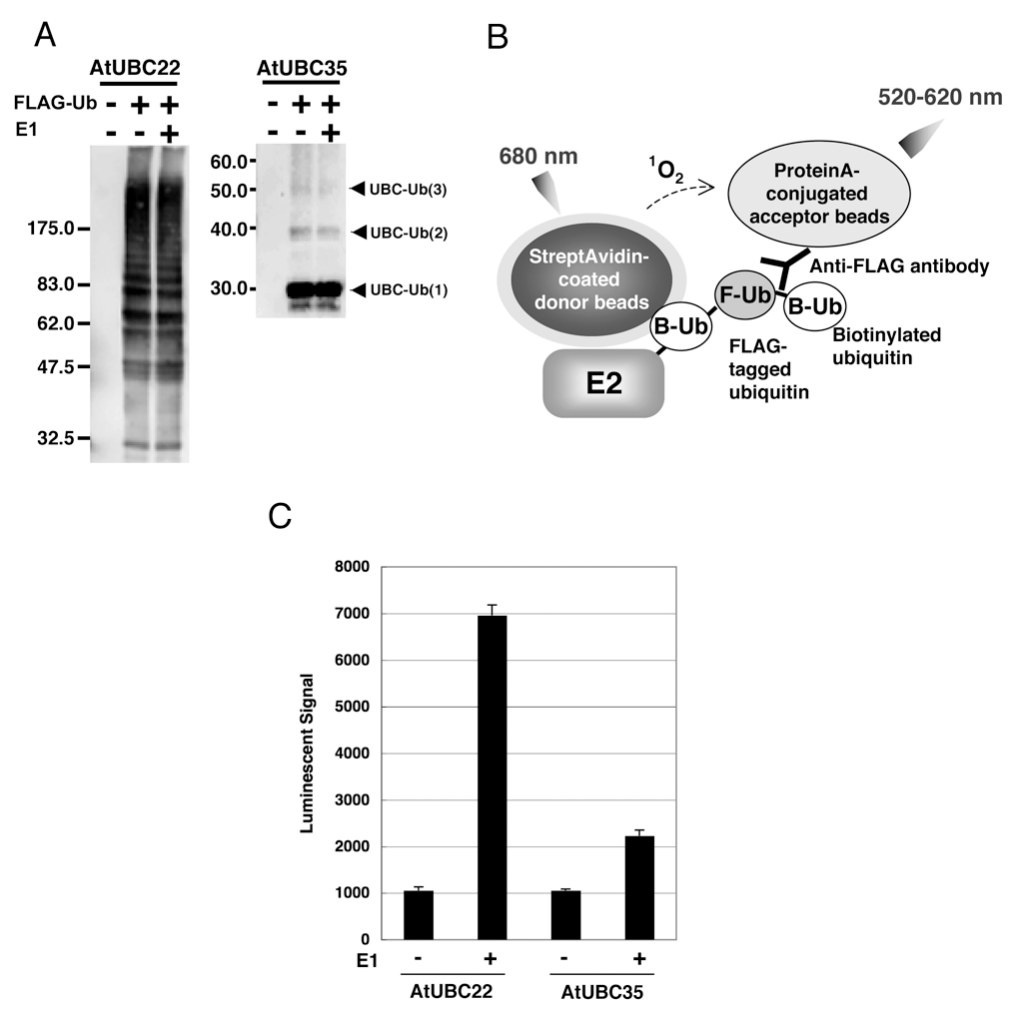
**Detection of E3-independent polyubiquitination of AtUBC22 by luminescent analysis**. A, Polyubiquitin chain on AtUBC22 but not on AtUBC35 was detected by immunoblot analysis. In this assay, polyubiquitination reaction was carried out with FLAG-tagged ubiquitin, and detected by immunoblot analysis using anti-FLAG antibody. B, Schematic diagram of detection of polyubiquitin chains by luminescent analysis. Protein A-conjugated acceptor beads and streptavidin-coated donor beads are bound to anti-FLAG antibody bound to FLAG-tagged ubiquitin and biotinylated E2, respectively, and these two beads are in closed proximity when polyubiquitin chain formed. Upon excitation 680 nm, a singlet oxygen is generated from the donor beads, and then transferred to the acceptor beads within 200 nm, and the singlet oxygen reacts the acceptor beads which in turn emits light at 520–620 nm. This light is measured by AlphaScreen kit and change to signal value. C, Polyubiquitin chain on purified recombinant E2 was detected by luminescent analysis in the presence (E1 +) or absence (E1 -) of exogenous E1. Error bars represent standard deviations from three independent experiments.

Recently, AtUBC22 (At5g05080) E2 protein has been shown to catalyze polyubiquitin chain formation without an E3 ligase, although AtUBC35 (At1g78870) E3-independent polyubiquitination activity could not be detected [16]. We employed AtUBC22 and AtUBC35 as model E2 proteins to develop a novel polyubiquitination assay. We have also demonstrated that addition of biotin ligase (BirA) and biotin to the wheat cell-free protein production system yields a single biotinylation on a target protein containing a biotin ligation site [22]. Using this method, biotinylated recombinant AtUBC22 and AtUBC35 were synthesized and, without purification from the translation mixture, the polyubiquitination reaction was performed on the crude recombinant protein. After the reaction, biotinylated AtUBC22 and AtUBC35 were purified using streptavidin-conjugated magnetic beads and the polyubiquitin chain was detected by immunoblot analysis. As shown in Fig 1A, AtUBC22 showed polyubiquitination, whereas AtUBC35 showed mainly monoubiquitination. Interestingly, both E2s still had activity in absence of exogenous E1 in polyubiquitin reaction mixture (Fig. 1A, middle lanes), suggesting that wheat cell-free system has high endogenous E1 activity.

While immunoblot analysis is an excellent detection method, it is not suitable for high-throughput detection of numerous polyubiquitination reactions. Initially, we attempted to use luminescent analysis, based on the AlphaScreen technology, to detect the polyubiquitination activity of AtUBC22 and AtUBC35. In principle, if a polyubiquitin chain is formed by FLAG-tagged and biotinylated ubiquitins, it will bring into proximity the streptavidin-coated donor bead (bound to biotin) and the protein A-conjugated acceptor bead (bound to anti-FLAG IgG), producing a luminescent signal (Fig. 1B). Considering that the wheat cell-free system has high endogenous E1 activity (Fig. 1A), it may also have endogenous E2 and E3 activity. In order to avoid formation of polyubiquitin chains by an endogenous wheat germ ubiquitin pathway, purified E2s were used in this assay. As shown in Fig 1C, high luminescent signal was observed in the presence of AtUBC22 in E1-dependent manner. In contrast, AtUBC35 showed low signal. The two luminescent signals were approximately consistent with immunoblot data that AtUBC22 and AtUBC35 have high and low polyubiquitination activities respectively, as demonstrated in Fig 1A. These results indicate that the luminescent method can detect polyubiquitin chain formation by using the two types of ubiquitins.

### Ubiquitination and Polyubiquitination Analyses of HECT-TypeE3 Ligases

**Figure 2:**
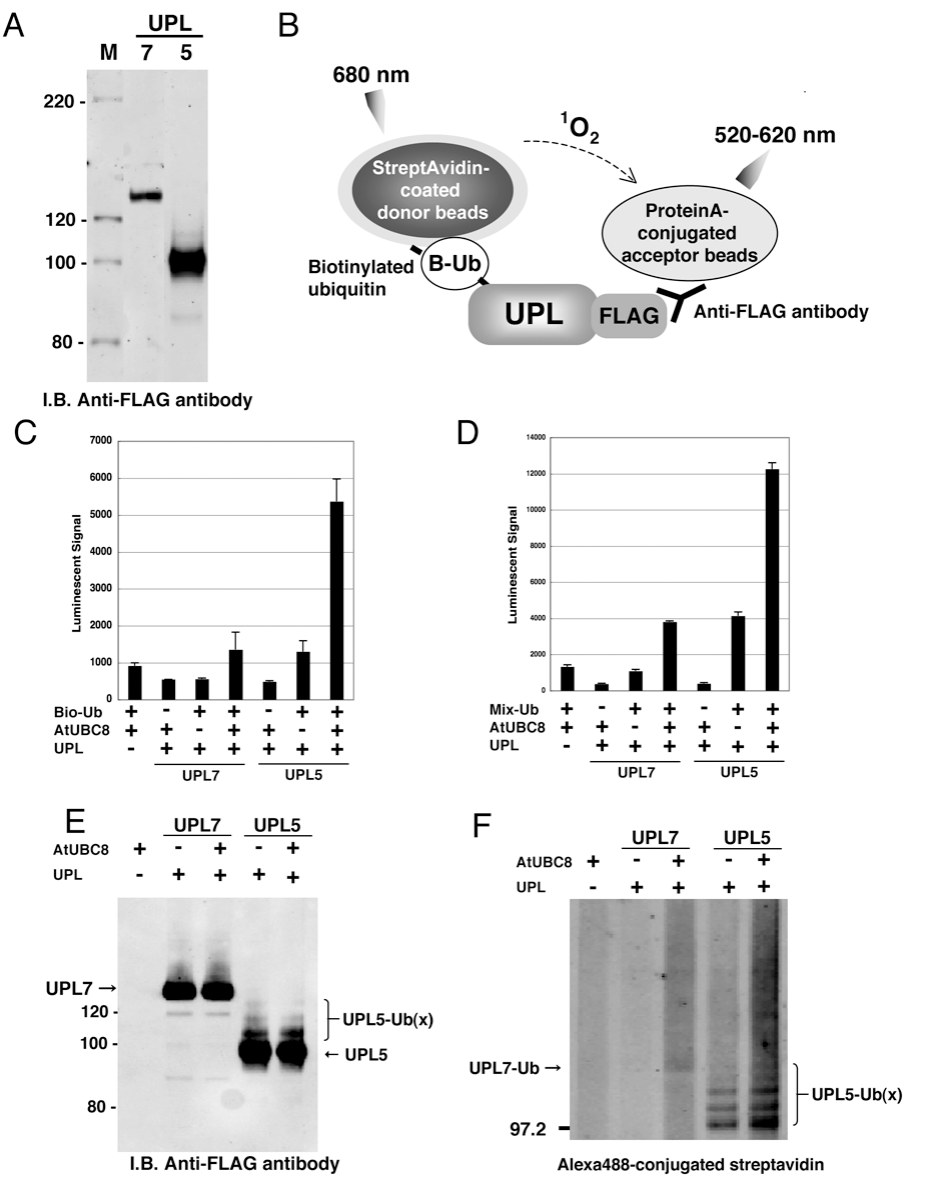
**Analysis of recombinant Arabidopsis HECT-type E3 ligases (UPL7 and UPL5)**. A, Production of FLAG-tagged recombinant UPL proteins was detected by immunoblot analysis. For analysis, 5 μl of crude recombinant UPL proteins were loaded, and detected by immunoblot analysis using anti-FLAG antibody. B, Schematic diagram of detection of ubiquitin-conjugation of UPLs by luminescent analysis. Protein A-conjugated acceptor beads and streptavidin-coated donor beads were bound to anti-FLAG antibody bound to FLAG-tagged recombinant UPLs and biotinylated ubiquitin, respectively, and detected by same principle and procedure described in Figure 1B. C, The ubiquitination of crude recombinant UPL7 and UPL5 was detected by luminescent analysis described in B. Bio-Ub means biotinylated ubiquitin. D, polyubiquitination of crude recombinant UPL7 and UPL5 was detected by luminescent analysis with anti-His antibody. Mix-Ub indicated the mixture of His-tagged and biotinylated ubiquitin. E and F, Mobility shift of UPLs (E) and formation of polyubiquitin chains (F) were detected by immunoblot using anti-FLAG antibody and Alexa488-conjugated streptavidin, respectively. The polyubiquitination reaction was done with FLAG-tagged recombinant UPLs in presence or absence of crude AtUBC8, and then recombinant UPLs were purified by anti-FLAG antibody-conjugated agarose. Error bars represent standard deviations from three independent experiments.

Polyubiquitination activity of E3 ligases activated by the step-wise E1 to E3 cascade is well documented [3]. We next attempted to reconstruct this cascade *in vitro *and to detect the E3-formed polyubiquitin chains using our luminescent method. Due to the size of HECT-type E3 ligases, ranging from 100 to 428 kDa in Arabidopsis, production of active protein by traditional expression methods may not be easy and biochemical analysis using only truncated recombinant protein has been carried out previously [23]. We attempted to produce full-length Arabidopsis HECT-type E3 ligase proteins using the wheat cell-free system and monitored ubiquitin-conjugation and polyubiquitination by luminescence. Two genes that encode Arabidopsis HECT-type E3 ligase, *UPL5 *and *UPL7 *[24], were analyzed in this study. We obtained UPL5 and UPL7 cDNA from the RAFL library and produced FLAG-tagged protein in the wheat cell-free system. Ubiquitination of FLAG-labeled UPLs (UPL-FLAGs) was investigated by both the luminescent and immunoblot methods. The successful production of the two recombinant HECT proteins was observed by immunoblot analysis (Fig. 2A) and used in the luminescence assay without purification. To detect ubiquitination of the HECT proteins, UPL-FLAGs and biotinylated ubiquitin were used. When biotinylated ubiquitin is conjugated to the UPL-FLAG, a high luminescent signal is obtained (Fig. 2B). As a result of the analysis, ubiquitin-conjugation of UPL5 was observed (Fig. 2C). In addition, polyubiquitin chains formed by UPLs were detected with the luminescence assay using His-tagged and biotinylated ubiquitin. To subtract polyubiquitin chain formation from endogenous E2 and E3 in wheat cell-free system, the assay was performed without recombinant UPL and only low signal was detected (Fig. 2D, "UPL-" lane). As expected, luminescent signal was observed in recombinant UPL5 and UPL7 (Fig. 2D). Although the luminescent signal of UPL7 was lower than that of UPL5, the signal was still two-fold higher than the endogenous background signal. These results were confirmed by immunoblot analysis that showed distinct mobility shifts of UPL5 (Fig. 2E) when detecting FLAG-tagged UPLs, and polyubiquitin chain formation of UPL5 monitoring Alexa488-conjugated streptavidin (Fig. 2F). Comparing the amount of polyubiquitin chain formation in absence of UPLs (Fig. 2F, "UPL-" lane), UPL7 formed weak but distinct polyubiquitin chains in presence of AtUBC8. These luminescent signals were consistent with immunoblot data. Interestingly, polyubiquitin chains were formed by UPL5 without supplementing exogenous E2 protein (Fig. 2D and 2F, "AtUBC8-" lane), suggesting that wheat germ extract has endogenous E2 activity as well as endogenous E1 activity. These data indicate that the wheat cell-free production system is able to produce high molecular weight proteins in functional forms and that our luminescence method can detect activity of HECT-type E3 ligases without purification. This is the first data showing that full length recombinant HECT-type E3s have ubiquitin-conjugating and polyubiquitination activity. Taken together, the luminescent method based on the wheat cell-free system could be useful for biochemical analysis of HECT-type E3 ligases.

### Detection of Polyubiquitin Chains by RING-Type CIP8 E3 Ligase

**Figure 3:**
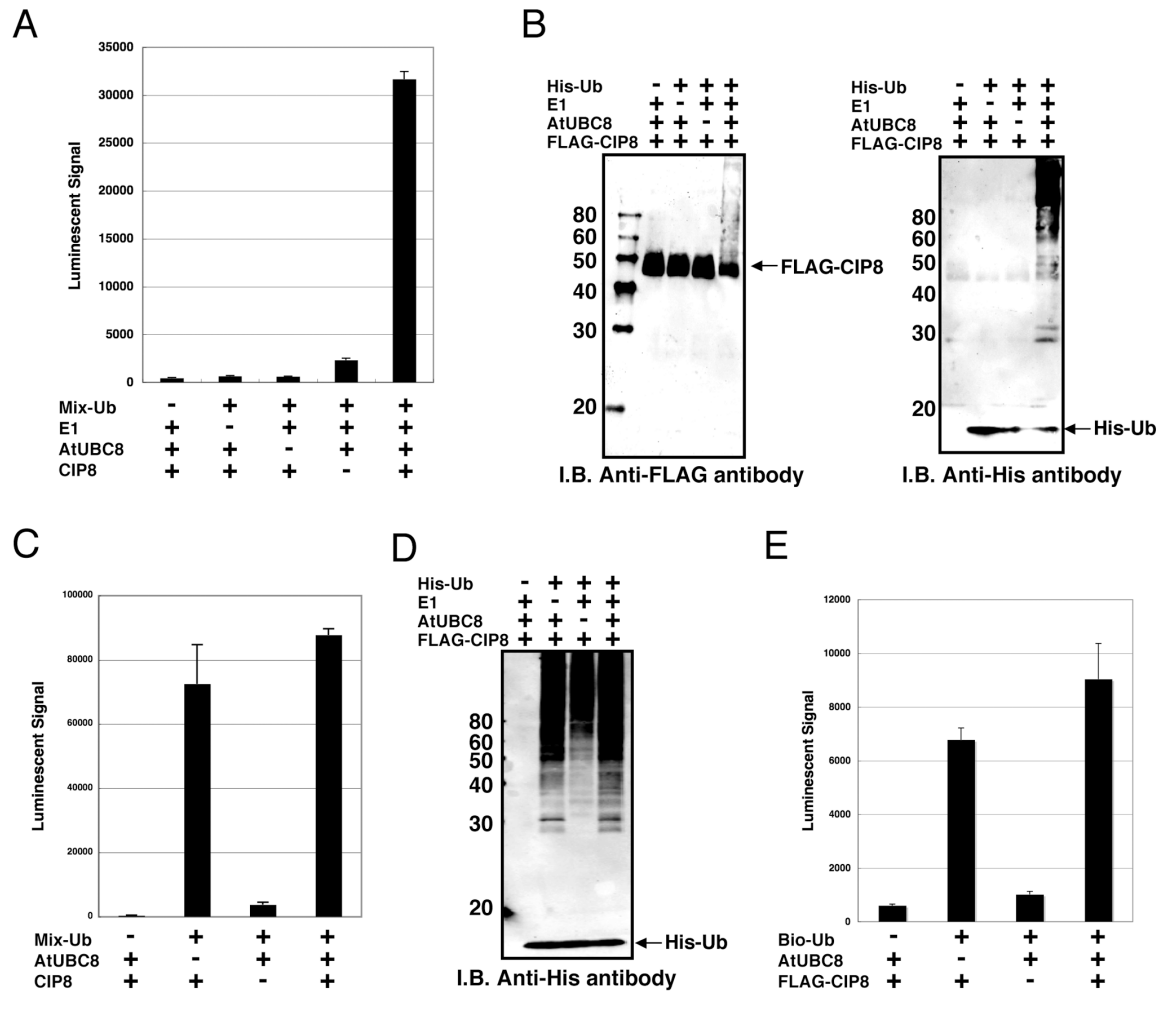
**Detection of polyubiquitination and self-ubiquitination of CIP8**. A to D, The polyubiquitination assay was carried out with purified (A and B) or crude recombinant CIP8 (C and D) and detected by luminescent analysis with anti-FLAG antibody (A and C) and immunoblot analysis (B and D). His-Ub or Mix-Ub indicate His-tagged ubiquitin or the mixture of FLAG-tagged and biotinylated ubiquitin, respectively. The polyubiquitination assay using luminescent analysis was carried out with recombinant CIP8 without tag in the presence or absence of ubiquitin related components indicated below each graph. E, Ubiquitination of crude recombinant CIP8 was observed by luminescent analysis with anti-FLAG antibody. The assay was carried out with or without biotinylated ubiquitin and crude AtUBC8 recombinant protein. Bio-Ub means biotinylated ubiquitin. Error bars represent standard deviations from three independent experiments.

It is reported that at least 469 predicted RING-type E3 ligases are encoded in the Arabidopsis genome [25]. Like the HECT-type E3, we attempted to express and carry out the functional analysis of the RING-type E3 ligases. In this study, we selected CIP8 as a model RING-type E3 ligase, which is reported to possess a RING finger motif and have typical features of an E3 ligase [26]. At first, polyubiquitination activity of purified CIP8 in presence or absence of exogenous E1 and purified E2 (AtUBC8) was investigated by luminescence. As shown in Fig 3A, luminescence analysis using His-tagged and biotinylated ubiquitin showed the polyubiquitination of purified CIP8 only when exogenous E1 and purified E2 were added to the reaction mixture. The CIP8-dependent polyubiquitination was confirmed by immunoblot analyses detecting both FLAG-CIP8 and His-tagged ubiquitin (Fig. 3B). On the other hand, luminescent analysis with crude CIP8 protein showed high polyubiquitination activity both in the presence or absence of purified E2 (Fig. 3C), and was confirmed by immunoblot analysis with crude protein (Fig. 3D). These data indicated that, like recombinant UPL5, crude CIP8 also utilized endogenous wheat extract E1 and E2 proteins, and therefore we could carry out the simple polyubiquitination analysis of E3 without addition of exogenous E1 and E2 proteins. Furthermore, immunoblot analysis detecting purified CIP8 (Fig. 3B) showed a mobility shift of FLAG-tagged CIP8 to higher molecular weights due to ubiquitination, whereas the mobility of the E2 was not altered (data not shown). This result indicates that the CIP8-dependent polyubiquitin chains might be elongated on CIP8 itself. This data is consistent with a recent report showing that TRIM5a, a typical RING-type E3 ligase in human, also undergoes self-ubiquitination, forming polyubiquitin chains on itself [27]. To clarify whether the mobility shift of CIP8 was concomitant with polyubiquitin chain formation resulting from self-ubiquitination, we tried to detect ubiquitination of CIP8 by the luminescent method using crude FLAG-CIP8 protein and biotinylated ubiquitin. The luminescent method clearly detected the binding of biotinylated ubiquitin to FLAG-tagged CIP8 both in the presence and absence of exogenous E2 (Fig. 3E). Similar to polyubiquitin formation, the ubiquitination of CIP8 also occurred without the addition of exogenous E2 protein (Fig. 3E, "AtUBC8-" lane). Taken together, these data demonstrate that the luminescent method could detect formation of RING-type CIP8-dependent polyubiquitin chains and self-ubiquitination of crude CIP8.

### Screening of RING-Type E3 Ligases Having Polyubiquitination Activity

Recent papers have reported that the polyubiquitin chain is an important biological regulator. Identification of activity and features of E3 ligases offers important information about the ubiquitin-dependent regulation system. Our luminescent method based on the wheat cell-free system produced a simple and high-sensitivity detection of CIP8-dependent polyubiquitin chains without any purification (Fig. 3C). Using these tools, we screened new E3 ligases for the ability to form polyubiquitin chains like CIP8.

**Figure 4:**
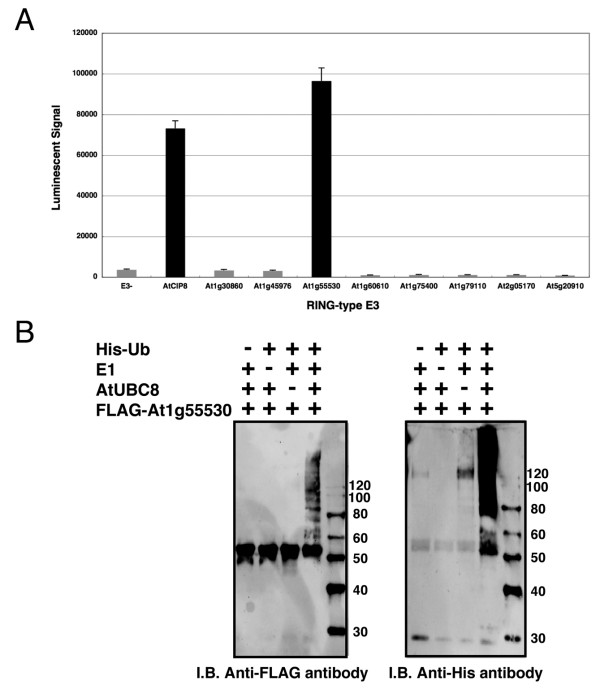
**Screening of RING-type E3 ligases having polyubiquitination activity**. A, Polyubiquitination reaction of crude recombinant E3 proteins was carried out with mixture of FLAG-tagged and biotinylated ubiquitins, and investigated by luminescent analysis with anti-FLAG antibody. B, Polyubiquitination activity of At1g55530 was confirmed by immunoblot analysis. The assay was carried out using purified recombinant AtUBC8 and At1g55530, and mobility shift of FLAG-tagged At1g55530 and polymer of His-ubiquitin were detected by immunoblot analysis using anti-FLAG and anti-His antibodies, respectively. Error bars represent standard deviations from three independent experiments.

The RING-type E3 ligases in Arabidopsis were divided into 30 subgroups based on domain structure, and CIP8 is categorized into subgroup 6 as it contains a coiled-coil domain [25]. Eight other RING-type E3s from subgroup 6 were selected for screening, and the simple polyubiquitination assay was carried out with FLAG-tagged and biotinylated ubiquitins, and the crude recombinant RING-type E3s without addition of exogenous E1 and E2. The screening result showed significant polyubiquitination activity of At1g55530, whereas other RING-E3 proteins were not active (Fig. 4A). Immunoblot analysis of purified recombinant At1g55530 confirmed the polyubiquitination activity and indicated that At1g55530 was self-ubiquitinated (Fig. 4B). The polyubiquitination activity of At1g55530 suggests that it may have a biological role for proteasome-mediated degradation like CIP8 [26]. These results show that the wheat cell-free protein expression system and the luminescent ubiquitination detection method could support functional high-throughput screening of E3 proteins.

### Analysis of the Wheat Cell-free Based Ubiquitination in the Presence of Proteasome Inhibitor

**Figure 5:**
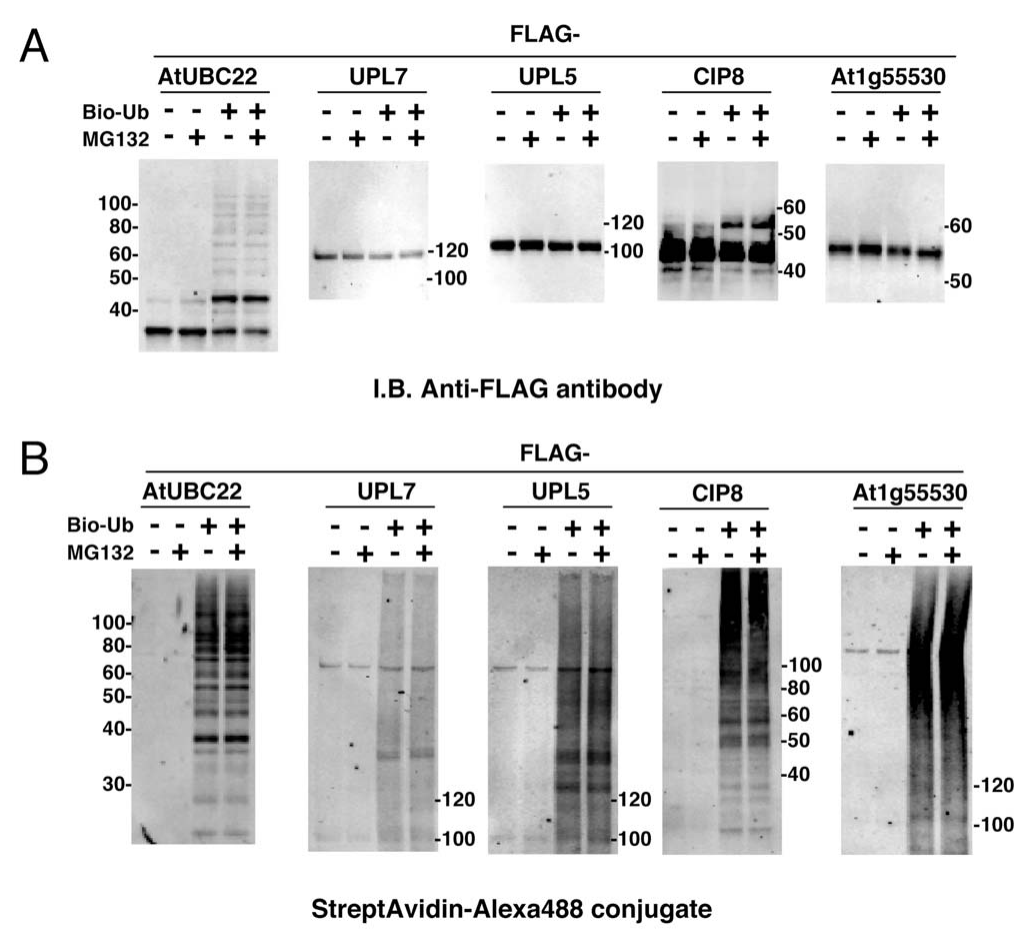
**Effect of proteasome inhibitor on stability of polyubiquitinated proteins**. Polyubiquitination assays of crude FLAG-tagged E2s and E3s were carried out in the presence or absence of biotinylated ubiquitin and 20 μM MG132. A, FLAG-tagged recombinant proteins were detected by immunoblot analysis using anti-FLAG antibody. B, Polyubiquitination chain formed by each recombinant protein was detected by Alexa488-conjugated streptavidin.

It is known that some cell extracts, such as rabbit reticulocyte or HeLa S-100 fraction, have 26S proteasome-dependent proteolytic activity [28,29]. Based on the presence of endogenous E1 and E2 ubiquitination and polyubiquitination in the wheat cell-free system, it is expected that the 26S proteasome activity will be very low (Fig. 2, 3 and 4). It was previously reported that the wheat germ extract had little 26S proteasome-dependent protein degradation activity [30]. Thus, we determined whether the wheat cell-free system contains active 26S proteasome. Using the crude recombinant proteins that formed polyubiquitin chains in this study, the polyubiquitination reaction was carried out in presence or absence of MG132, and accrual of the polyubiquitinated recombinant proteins and its polyubiquitin chain was estimated. As shown in Fig 5, the amounts of UBC22, UPL5, UPL7 and At1g55530 (Fig. 5A) and of its polyubiquitin chains (Fig. 5B) were hardly altered by MG132 treatment. This result indicates that the proteolytic activity of the 26S proteasome in the wheat cell-free system was below the detection level. Thus, the wheat cell-free system could be suitable for ubiquitination analysis.

## Discussion

The ubiquitin signal is an important protein modification in eukaryotes. Binding of a single ubiquitin to a target protein, mono-ubiquitination, is essential for membrane trafficking, protein functions and protein-protein interaction [7]. As for polyubiquitination, both Lys-48- and Lys-63-linked polyubiquitin chains have been well characterized in mammals and yeast. Lys-48 linked chains cause proteolysis of target proteins [6], and Lys-63 linked chains regulate signal transduction such as cellular localization of protein or protein-protein interactions [7]. In mammals, the multi-functional activities of NF-κB are regulated by the Lys-63 linked chain [31]. In plants, the function of the Lys-63 linked chain is still obscure. However, Arabidopsis E2 and its variants promote formation of the Lys-63 linked chain [32], suggesting that the Lys-63 linked chain in plant cells might also function similar to animal cells. Hence, comprehensive analysis of the ubiquitin-related plant proteins would open a door for elucidation of the plant ubiquitin pathway. In this study, we developed a simple and highly sensitive ubiquitination assay method by combination of the wheat cell-free protein synthesis system and luminescent detection. In general, *in vivo *protein production requires many time-consuming steps such as vector construction, cell culture and purification to obtain the recombinant protein. In contrast, this cell-free based luminescence method could analyze a large amount of ubiquitin reactions without these steps.

Using this method, we conveniently detected polyubiquitin chain formation of E2 and E3s by using two tagged ubiquitins (Fig. 1, 2, 3 and 4). The result of polyubiquitination analysis of the E2s obtained from luminescent-based detection method was verified by immunoblot analysis (Fig. 1). Our analysis also produced recombinant protein of HECT-type E3 ligases without truncation and detected their ubiquitin-conjugation and polyubiquitination activity by luminescent analysis (Fig. 2C and 2D). The ubiquitin-conjugation of UPL5 was not observed when a reductant was added to the reaction (data not shown), suggesting that UPL5 formed a thioester bond with ubiquitin. In addition, the model RING-type E3 CIP8 possessed high polyubiquitin formation activity without substrate, consistent with what was reported previously [26]. Crude recombinant CIP8 formed polyubiquitin chains in the absence of exogenous E1 and E2 (Fig. 3C and 3D), suggesting that the wheat cell-free system might include enough endogenous E1 and E2 activity. It was reported that wheat germ extracts have only a partial ubiquitin pathway [30]. Although the process to isolate wheat germ extract is different from the conventional methods [33], this report strongly supports the existence of endogenous ubiquitin pathway in our wheat cell-free system. Indeed, luminescent analysis using crude recombinant protein showed slight polyubiquitin chain formation even in absence of recombinant E3 (Fig. 2D, Fig. 3C and Fig. 4A, "E3-" lane), indicating that wheat cell-free system might include not only E1 and E2, but E3s or other factors that accelerates the polyubiquitin chain formation. Further, quantitative immunoblot analysis using anti-ubiquitin antibody showed that free ubiquitin was also present in wheat germ extract at a concentration of at least 10 nM (data not shown). This is similar to the ubiquitin concentration supplied in the *in vitro *assay. Although we developed a convenient screening method to detect E3 activity in this study, removal of the endogenous ubiquitin and ubiquitin related components such as E1, E2 and E3, would yield a more sensitive assay. However, wheat cell-free system does not have 26S proteasome proteolytic activity (Fig. 5), indicating that using crude recombinant protein is sufficient for *in vitro *ubiquitination assays.

By using this method, we found that a previously uncharacterized RING type E3, At1g55530, possessed high polyubiquitination activity without exogenous E1 and E2 proteins (Fig. 4). This result suggested that the method developed here is expected to find the activity of other unknown E3 ligases such as At1g55530. Despite having only 32% sequence similarity, the E3s CIP8 and At1g55530 showed similar biochemical functions. Polyubiquitin chains formed by CIP8 and At1g55530 elongated on themselves, while another report showed that polyubiquitin chains were formed on E2 before transferring them to substrates [34]. This reflects that the pattern of polyubiquitin chain formation differs between individual E3s and that the detailed mechanisms are still unknown. These studies suggest the importance of functional analysis using active recombinant proteins. Although we developed a simple screen using crude recombinant E3s in absence of exogenous E1 and E2 (Fig. 4), this method could not detect the activity of some E3 ligases that were unable to utilize endogenous ubiquitination components in wheat cell-free system. The polyubiquitination activity of At5g20910 recombinant protein, expressed in *E. coli *in the presence of AtUBC8 [25], was not active in our *in vitro *system (Fig. 4A), suggesting that in some cases exogenous E2 and/or other components are necessary additions. Such modifications to the ubiquitination assays detailed here would help elucidate the biochemical features of E3s (e.g., addition of recombinant E2s to reaction mixture could give us further information about the E2–E3 specificity, and of other E3 components would lead to the elucidation of structure of complex type E3 ligase such as SCF).

## Conclusion

In this study, we found that the wheat cell-free system was an excellent expression system to produce recombinant protein efficiently and to carry out *in vitro *ubiquitination assays without the interference of proteolytic activity. Coupled with luminescent analysis, detection of these ubiquitin reactions in the crude translation reaction mixture was possible. Thus, this method should be helpful for solving the complicated ubiquitin pathway in plant.

## Methods

### Construction of DNA Templates for Transcription

We used RAFL as templates. DNA templates of E2s and E3s for transcription were constructed by "Split-Primer" PCR as described previously [17]. Primers used in this study are summarized in Additional file 1. The first round of PCR was performed on each cDNA template using 10 nM of each of the following primers: a target protein specific primer (5'-CCACCCACCACCACCAatgnnnnnnnnnnnnnnnn-3'; lowercase indicates the 5'-coding region of the target gene) and the AODA2306 primer. Then, a second round of PCR was carried out to construct the templates for protein synthesis using a portion (5 μl) of the first PCR mix, 100 nM SPu primer, 100 nM AODA2303 primer and 1 nM deSP6E02 primer. GST tags were used according to the methods we described previously [17]. The transcription templates of two HECT-type E3 ligases, UPL7 and UPL5, were generated as C-terminal FLAG-tagged proteins using the Gateway System^® ^(Invitrogen, Carlsbad, CA, USA). Briefly, the ORF sequences of UPL7 and UPL5 were amplified by PCR with sense and antisense primers containing attB1 and FLAG-attB2 sequences, respectively. According to the manufacturer's instructions (Invitrogen), these DNA fragments were subcloned into pDONR221 vector by BP reaction and then inserted into the Gateway-based pEU vector (pEU-E01-GW) by LR reaction. Using these recombinant vectors as templates, PCR was carried out with 100 nM SPu primer and 100 nM AODA2303 primer and used as transcription templates.

### Cell-free Protein Synthesis

*In vitro *transcription and cell-free protein synthesis were performed as described [18]. Transcript was made from each of the DNA templates mentioned above using the SP6 RNA polymerase. The synthetic mRNAs were then precipitated with ethanol and collected by centrifugation using a Hitachi R10H rotor. Each mRNA (usually 30–35 μg) was washed and transferred into a translation mixture. The translation reaction was performed in the bilayer mode [35] with slight modifications. The translation mixture that formed the bottom layer consisted of 60 A260 units of the wheat germ extract (CellFree Sciences, Yokohama, Japan) and 2 μg creatine kinase (Roche Diagnostics K. K., Tokyo, Japan) in 25 μl of SUB-AMIX^® ^(CellFree Sciences). The SUB-AMIX^® ^contained (final concentrations) 30 mM Hepes/KOH at pH 8.0, 1.2 mM ATP, 0.25 mM GTP, 16 mM creatine phosphate, 4 mM DTT, 0.4 mM spermidine, 0.3 mM each of the 20 amino acids, 2.7 mM magnesium acetate, and 100 mM potassium acetate. SUB-AMIX^® ^(125 μl) was placed on the top of the translation mixture, forming the upper layer. After incubation at 16°C for 15 h, the synthesized proteins were confirmed by SDS-PAGE. For biotin labeling, 1 μl of crude biotin ligase (BirA) produced by the wheat cell-free expression system was added to the bottom layer, and 0.5 μM (final concentration) of D-biotin (Nacalai Tesque, Inc., Kyoto, Japan) was added to both upper and bottom layers, as described previously [22].

### Purification of E2 and E3 Proteins

Purification of GST-tagged protein was carried out according to the procedure described previously [36] with slight modification. Crude GST-tagged recombinant protein (450 μl) produced by the cell-free reaction was precipitated with glutathione sepharose™ 4B (GE Healthcare, Buckinghamshire, UK). The recombinant proteins were eluted with PBS buffer containing 0.1 U of AcTEV protease (Invitrogen) in order to cleave the GST tag from the protein.

### Detection of Polyubiquitination by the Luminescent Method

*In vitro *polyubiquitination assays were carried out in a total volume of 15 μl consisting of 20 mM Tris-HCl pH 7.5, 0.2 mM DTT, 5 mM MgCl_2_, (10 μM zinc acetate in the assays for RING-type E3s only), 3 mM ATP, 1 mg/ml BSA, 25 nM biotinylated ubiquitin, 25 nM FLAG-tagged ubiquitin, 1 μl of recombinant E2 (purified or crude) and 1 μl of recombinant E3 (purified or crude) in the presence or absence of 0.05 μM rabbit E1 (Boston Biochem, Cambridge, MA, USA) at 30°C for 1 hr in a 384-well Optiplate (PerkinElmer, Boston, MA, USA). In accordance with the AlphaScreen IgG (ProteinA) detection kit (Perkin Elmer) instruction manual, 10 μl of detection mixture containing 20 mM Tris-HCl pH 7.5, 0.2 mM DTT, 5 mM MgCl_2_, 5 μg/ml Anti-FLAG antibody (Sigma-Aldrich, St. Louis, MO, USA), 1 mg/ml BSA, 0.1 μl streptavidin-coated donor beads and 0.1 μl anti-IgG acceptor beads were added to each well of the 384 Optiplate followed by incubation at 23°C for 1 hr. Luminescence was analyzed by the AlphaScreen detection program.

### Detection of Ubiquitinated E2 by Immunoblot Analysis

Crude biotinylated recombinant E2 proteins (40 μl) were used for the ubiquitin-conjugating assay in a total reaction volume of 50 μl containing 20 mM Tris-HCl pH 7.5, 0.2 mM DTT, 5 mM MgCl_2_, 3 mM ATP and 4 μM FLAG-tagged ubiquitin (Sigma) for 3 hr at 30°C. The reaction products were purified by Streptavidin Magnesphere Paramagnetics particles (Promega, Madison, WI, USA). After washing the beads with PBS buffer, recombinant E2s were boiled in 15 μl of SDS sample buffer containing 50 mM Tris-HCl pH 6.8, 2% SDS, 10% glycerol and 0.2% bromophenol blue, and then separated from the magnet beads. The proteins were separated by SDS-PAGE and transferred to PVDF membrane (Millipore Bedford, MA, USA) according to standard procedures. The blots were detected by the ECL plus detection system (GE Healthcare) with anti-FLAG antibody (Sigma) according to the manufacturer's procedure.

### Detection of Polyubiquitination by the Immunoblot Analysis

For polyubiquitination of HECT-type E3 ligases, crude FLAG-tagged UPL recombinant protein (20 μl) was ubiquitinated in a total reaction volume of 50 μl consisting of 20 mM Tris-HCl pH 7.5, 0.2 mM DTT, 5 mM MgCl_2_, 3 mM ATP, 4 μM biotinylated ubiquitin and 20 μl of crude recombinant AtUBC8 for 3 hr at 30°C. Then, recombinant UPL protein was gathered by anti-FLAG M2 agarose (Sigma). After washing the agarose with PBS buffer, the recombinant UPL protein was boiled in 15 μl of SDS sample buffer and then separated from beads by centrifugation. For polyubiquitination of RING-type E3 ligases, the assay was carried out in 10 μl of reaction mixture containing 20 mM Tris-HCl pH 7.5, 0.2 mM DTT, 5 mM MgCl_2_, 10 μM zinc acetate, 3 mM ATP, 1 mg/ml BSA, 4 μM FLAG- or His-tagged ubiquitin, 1 μl of purified or crude recombinant E2 and 1 μl of purified or crude recombinant E3 at 30°C for 3 hr. Then, 5 μl of three-fold concentrated SDS sample buffer was added to the reaction mixture and boiled for 5 min. Proteins were separated by SDS-PAGE and transferred to Hybond-LFP PVDF membrane (GE Healthcare) according to standard procedures. Immunoblot analysis was carried out with anti-FLAG antibody (Sigma) or anti-His antibody (GE Healthcare) according to the procedure described above. When detecting biotinylated ubiquitin, blots were treated with 5 μg/ml Alexa488-conjugated streptavidin (Invitrogen) in PBS buffer. After washing with PBS containing 0.1% Tween-20, the blot was analyzed by a Typhoon Imager (GE Healthcare) using the 532 nm laser and 526 emission filters.

### Polyubiquitination Assay with 26S Proteasome Inhibitor

Polyubiquitination reaction was carried out as same procedure described above except addition of MG132 (Calbiochem, San Diego, CA, USA) at a final concentration of 20 μM to reaction mixture. Then, the protein on blot was detected by immunoblot analysis with anti-FLAG antibody or Alexa488-conjugated streptavidin.

## Authors' contributions

HT conceived the study and performed the experiments, and contributed to writing the manuscript. MS and KS provided RAFL cDNA clones. AN conceived the study. YE conceived the study and supervised the work. TS conceived and designed the study, supervised the work and contributed to writing the manuscript.

## Supplementary Material

Additional file 1 **AGI code of Arabidopsis genes and primer sequences used in this study.** Click here for file

## References

[B1] Bai C, Sen P, Hofmann K, Ma L, Goebl M, Harper JW, Elledge SJ (1996). SKP1 Connects Cell Cycle Regulators to the Ubiquitin Proteolysis Machinery through a Novel Motif, the F-Box. Cell.

[B2] Chen Z, Hagler J, Palombella VJ, Melandri F, Scherer D, Ballard D, Maniatis T (1995). Signal-induced site-specific phosphorylation targets IκBα to the ubiquitin-proteasome pathway. Genes Dev.

[B3] Pickart CM (2001). Mechanisms underlying ubiquitination. Annu Rev Biochem.

[B4] Smalle J, Vierstra RD (2004). The ubiquitin 26S proteasome proteolytic pathway. Annu Rev Plant Biol.

[B5] Borden KL (2000). RING domains: master builders of molecular scaffolds?. J Mol Biol.

[B6] Glickman MH, Ciechanover A (2002). The ubiquitin-proteasome proteolytic pathway: destruction for the sake of construction. Physiol Rev.

[B7] Schnell JD, Hicke L (2003). Non-traditional functions of ubiquitin and ubiquitin-binding proteins. J Biol Chem.

[B8] Hofmann RM, Pickart CM (1999). Noncanonical MMS2-encoded ubiquitin-conjugating enzyme functions in assembly of novel polyubiquitin chains for DNA repair. Cell.

[B9] Yin XJ, Volk S, Ljung K, Mehlmer N, Dolezal K, Ditengou F, Hanano S, Davis SJ, Schmelzer E, Sandberg G, Teige M, Palme K, Pickart C, Bachmair A (2007). Ubiquitin lysine 63 chain forming ligases regulate apical dominance in Arabidopsis. Plant Cell.

[B10] Hicke L (2001). A new ticket for entry into budding vesicles – ubiquitin. Cell.

[B11] Vierstra RD (2003). The ubiquitin/26S proteasome pathway, the complex last chapter in the life of many plant proteins. Trends Plant Sci.

[B12] Nelson DC, Lasswell J, Rogg LE, Cohen MA, Bartel B (2000). FKF1, a Clock-Controlled Gene that Regulates the Transition to Flowering in Arabidopsis. Cell.

[B13] Osterlund MT, Hardtke CS, Wei N, Deng XW (2000). Targeted destabilization of HY5 during light-regulated development of Arabidopsis. Nature.

[B14] Stone SL, Williams LA, Farmer LM, Vierstra RD, Callis J (2006). KEEP ON GOING, a RING E3 ligase essential for Arabidopsis growth and development, is involved in abscisic acid signaling. Plant Cell.

[B15] Rosebrock TR, Zeng L, Brady JJ, Abramovitch RB, Xiao F, Martin GB (2007). A bacterial E3 ubiquitin ligase targets a host protein kinase to disrupt plant immunity. Nature.

[B16] Kraft E, Stone SL, Ma L, Su N, Gao Y, Lau OS, Deng XW, Callis J (2005). Genome analysis and functional characterization of the E2 and RING-type E3 ligase ubiquitination enzymes of Arabidopsis. Plant Physiol.

[B17] Sawasaki T, Ogasawara T, Morishita R, Endo Y (2002). A cell-free protein synthesis system for high-throughput proteomics. Proc Natl Acad Sci USA.

[B18] Sawasaki T, Gouda MD, Kawasaki T, Tsuboi T, Tozawa Y, Takai K, Endo Y (2005). The wheat germ cell-free expression system: methods for high-throughput materialization of genetic information. Methods Mol Biol.

[B19] Kobayashi T, Kodani Y, Nozawa A, Endo Y, Sawasaki T (2008). DNA-binding profiling of human hormone nuclear receptors via fluorescence correlation spectroscopy in a cell-free system. FEBS Lett.

[B20] Seki M, Narusaka M, Kamiya A, Ishida J, Satou M, Sakurai T, Nakajima M, Enju A, Akiyama K, Oono Y, Muramatsu M, Hayashizaki Y, Kawai J, Carninci P, Itoh M, Ishii Y, Arakawa T, Shibata K, Shinagawa A, Shinozaki K (2002). Functional annotation of a full-length Arabidopsis cDNA collection. Science.

[B21] Kus B, Gajadhar A, Stanger K, Cho R, Sun W, Rouleau N, Lee T, Chan D, Wolting C, Edwards A, Bosse R, Rotin D (2005). A high throughput screen to identify substrates for the ubiquitin ligase Rsp5. J Biol Chem.

[B22] Sawasaki T, Kamura N, Matsunaga S, Saeki M, Tsuchimochi M, Morishita R, Endo Y (2008). Arabidopsis HY5 protein functions as a DNA-binding tag for purification and functional immobilization of proteins on agarose/DNA microplate. FEBS Lett.

[B23] Bates PW, Vierstra RD (1999). UPL1 and 2, two 405 kDa ubiquitin-protein ligases from Arabidopsis thaliana related to the HECT-domain protein family. Plant J.

[B24] Downes BP, Stupar RM, Gingerich DJ, Vierstra RD (2003). The HECT ubiquitin-protein ligase (UPL) family in Arabidopsis: UPL3 has a specific role in trichome development. Plant J.

[B25] Stone SL, Hauksdóttir H, Troy A, Herschleb J, Kraft E, Callis J (2005). Functional analysis of the RING-type ubiquitin ligase family of Arabidopsis. Plant Physiol.

[B26] Hardtke CS, Okamoto H, Deng XW (2002). Biochemical evidence for ubiquitin ligase activity of the Arabidopsis COP1 interacting protein 8 (CIP8). Plant J.

[B27] Yamauchi K, Wada K, Tanji K, Tanaka M, Kamitani T (2008). Ubiquitination of E3 ubiquitin ligase TRIMa and its potential role. FEBS J.

[B28] Waxman L, Fagan JM, Goldberg AL (1987). Demonstration of two distinct high molecular weight proteases in rabbit reticulocytes, one of which degrades ubiquitin conjugates. J Biol Chem.

[B29] Chen ZJ, Parent L, Maniatis T (1996). Site-Specific Phosphorylation of IκBα by a Novel Ubiquitination-Dependent Protein Kinase Activity. Cell.

[B30] Hatfield PM, Vierstra RD (1989). Ubiquitin-dependent proteolytic pathway in wheatgerm: Isolation of multiple forms of ubiquitin-activating enzyme, E1. Biochemistry.

[B31] Wu CJ, Conze DB, Li T, Srinivasula SM, Ashwell JD (2006). Sensing of Lys 63-linked polyubiquitination by NEMO is a key event in NF-κB activation. Nature Cell Biol.

[B32] Yin XJ, Volk S, Ljung K, Mehlmer N, Dolezal K, Ditengou F, Hanano S, Davis SJ, Schmelzer E, Sandberg G, Teige M, Palme K, Pickart C, Bachmaira A (2007). Ubiquitin lysine 63 chain-forming ligases regulate apical dominance in Arabidopsis. Plant Cell.

[B33] Madin K, Sawasaki T, Ogasawara T, Endo Y (2000). A highly efficient and robust cell-free protein synthesis system prepared from wheat embryos: Plants apparently contain a suicide system directed at ribosomes. Proc Natl Acad Sci USA.

[B34] Li W, Tu D, Brunger AT, Ye Y (2007). A ubiquitin ligase transfers preformed polyubiquitin chains from a conjugating enzyme to a substrate. Nature.

[B35] Sawasaki T, Hasegawa Y, Tsuchimochi M, Kamura N, Ogasawara T, Kuroita T, Endo Y (2002). A bilayer cell-free protein synthesis system for high-throughput screening of gene products. FEBS Lett.

[B36] Masaoka T, Nishi M, Ryo A, Endo Y, Sawasaki T (2008). The wheat germ cell-free based screening of protein substrates of calcium/calmodulin-dependent protein kinase II delta. FEBS Lett.

